# Nano-adjuvants enhance the immunogenicity and safety of infectious bursal disease virus vaccines: a comprehensive review

**DOI:** 10.3389/fimmu.2026.1805229

**Published:** 2026-05-15

**Authors:** Tayyab Ali Asif, Pengchao Ji, Xinxin Qiao, Qun Yang, Dawei Jiang, Gaiping Zhang

**Affiliations:** 1 College of Veterinary Medicine, Henan Agricultural University, Zhengzhou, China; 2 International Joint Research Center of National Animal Immunology, College of Veterinary Medicine, Henan Agricultural University, Zhengzhou, China; 3 Longhu Laboratory, Zhengzhou, China

**Keywords:** IBDV, IBDV vaccines, immunity, nano-adjuvant, nanotechnology

## Abstract

Infectious bursal disease virus (IBDV) remains a major global threat to poultry, causing severe immunosuppression and substantial economic losses. Existing vaccines live-attenuated, inactivated, and subunit provide incomplete protection and face challenges of safety, antigenic mismatch, and repeated dosing requirements. Nano-adjuvant technologies offer a transformative solution, enhancing vaccine immunogenicity, stability, and targeted delivery. Platforms include lipid nanoparticles, polymeric systems, virosomes, mesoporous silica, polysaccharides, and nanoemulsions elicit robust humoral and cellular immune responses, promote Th1/Th2 balance, and enable dose-sparing strategies. Toll-like receptor agonist-loaded nano-adjuvants further amplify dendritic cell activation, antigen presentation, and durable memory responses. Virus-like particles and virosomes structurally mimic IBDV antigens, enhancing immunogenicity without the risks of viral replication. Rational integration of precise antigen selection, multifunctional nano-adjuvants, and advanced delivery approaches promises next-generation vaccines capable of broad, long-lasting protection against very virulent and emerging IBDV variants. These innovations have the potential to revolutionize poultry vaccination, offering safer, more effective, and globally deployable strategies to safeguard poultry health and productivity.

## Introduction

Infectious bursal disease virus (IBDV) is an acute, highly contagious pathogen belonging to the genus *Avibirnavirus* within the family *Birnaviridae* (1). Infectious bursal disease (IBD) remains a major global concern, as it is present 80% of the World Organization for Animal Health (OIE) member countries (2, 3). IBDV specifically affects young chicken worldwide. Outbreaks of IBD occur at different frequencies in Canada (North America), the USA, Morocco (Northwestern Africa), India, Japan, the United Kingdom, China, and Bangladesh (4–7). A mortality rate of up to 100% in specific pathogen-free (SPF) chickens was observed in Europe and subsequently 50-60% in laying hens. Similarly, very virulent infectious bursal disease virus (vvIBDV) causes 25-30% death rate in broilers was reported in Japan, and 70% mortality rate has been observed in Pakistan (8, 9). Two Chinese vvIBDV strains including HeB10 and HLJ0504 caused 40% and 52% mortality respectively (10). In Canada, each year direct losses of 3.9 million kg meat due to this infectious disease (11). IBD has a negative impact on the productivity of indigenous chickens which possess desirable characteristics such as disease resistance, thermo tolerance, meat laver and better egg production, high fertility, hard eggshells, and hatchability (12, 13). Therefore, efficient strategies are required to improve vaccine potency and efficacy.

**Figure 1:**
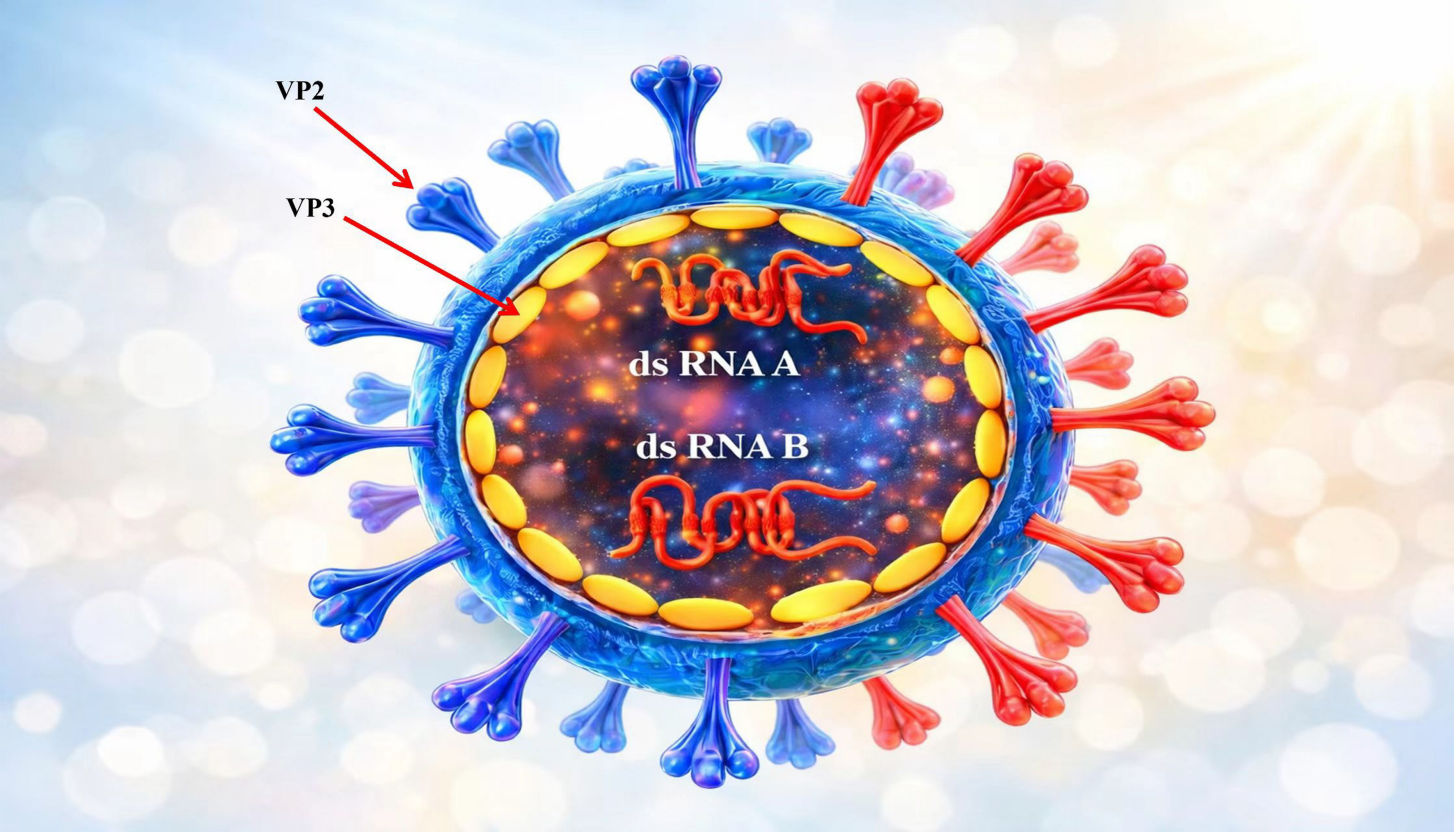
Molecular structure and capsid organization of IBDV (14).

The IBDV is a non-enveloped, single-shelled virion with icosahedral symmetry and a diameter ranging from 55 to 65 nm (13). Incomplete virus particles have lower density levels. The capsid symmetry is curled, with a triangulation number of T = 13. Although IBDV was previously believed to have a dextro-handed capsid symmetry, recent structural analyses have demonstrated that it adopts a conventional *laevo* icosahedral geometry (13). The structure of IBDV is shown in **Figure 1** (14).

**Figure 2:**
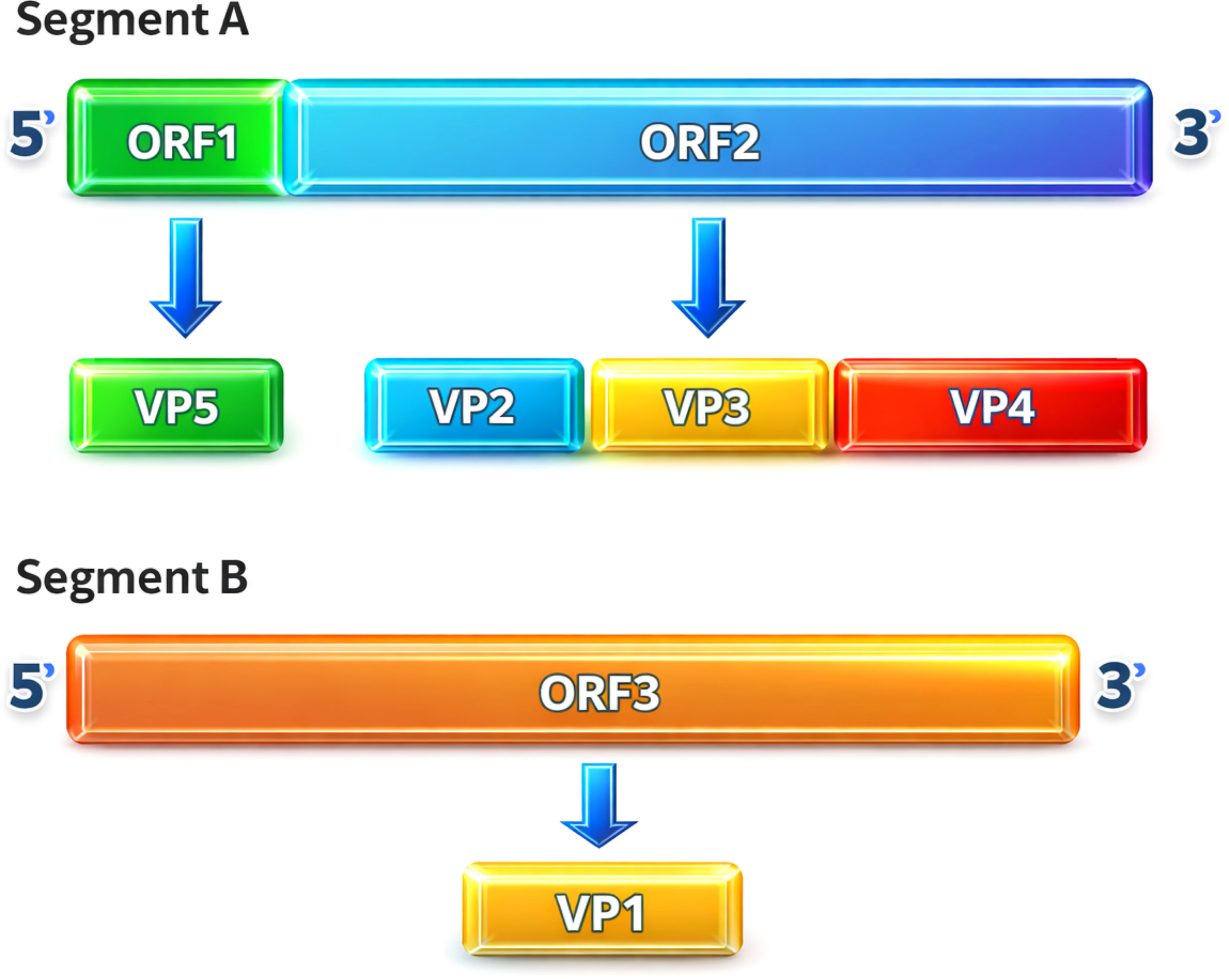
Genomic organization of IBDV (20).

The IBDV genome comprises two segments of double-stranded RNA, a hallmark of *birnaviruses* (15). Segment A (3.4 kb) contains two open reading frames: a large polycistronic ORF that undergoes autoproteolytic processing to generate the mature structural proteins VP2 and VP3 and the protease VP4, and a smaller ORF encoding VP5 (16, 17). Segment B (2.8 kb) encodes VP1, the viral RNA-dependent RNA polymerase (18). The viral capsid is primarily composed of the major structural proteins VP2 and VP3, with VP1, VP4, and VP5 classified as minor viral proteins that contribute to replication, virulence, and host immune modulation (19). IBDV genome organization shown on **Figure 2** (20).

**Figure 3:**
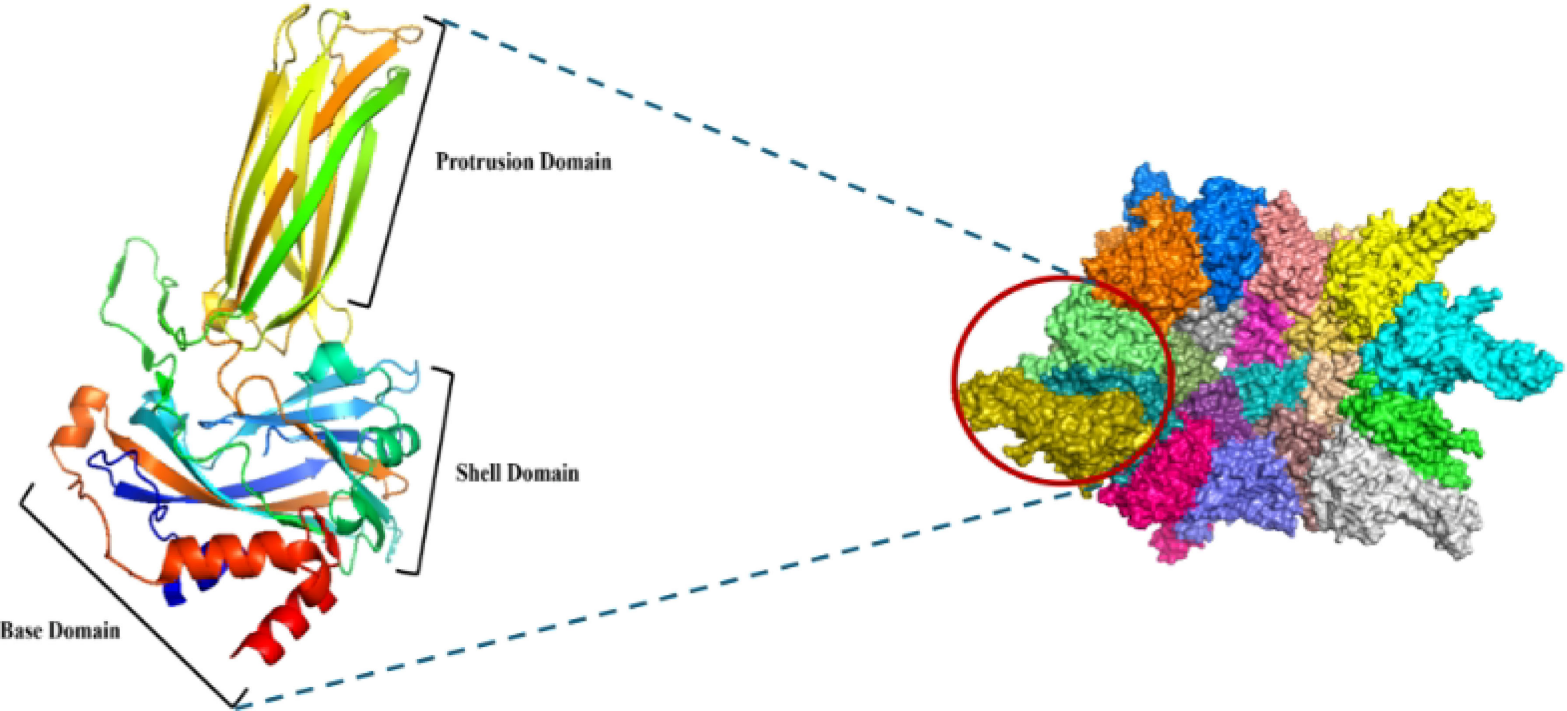
Self-assembly of IBDV VP2 monomers into virus-like particles (VLPs) (PDB code: 2DF7) (22).

**Figure 4:**
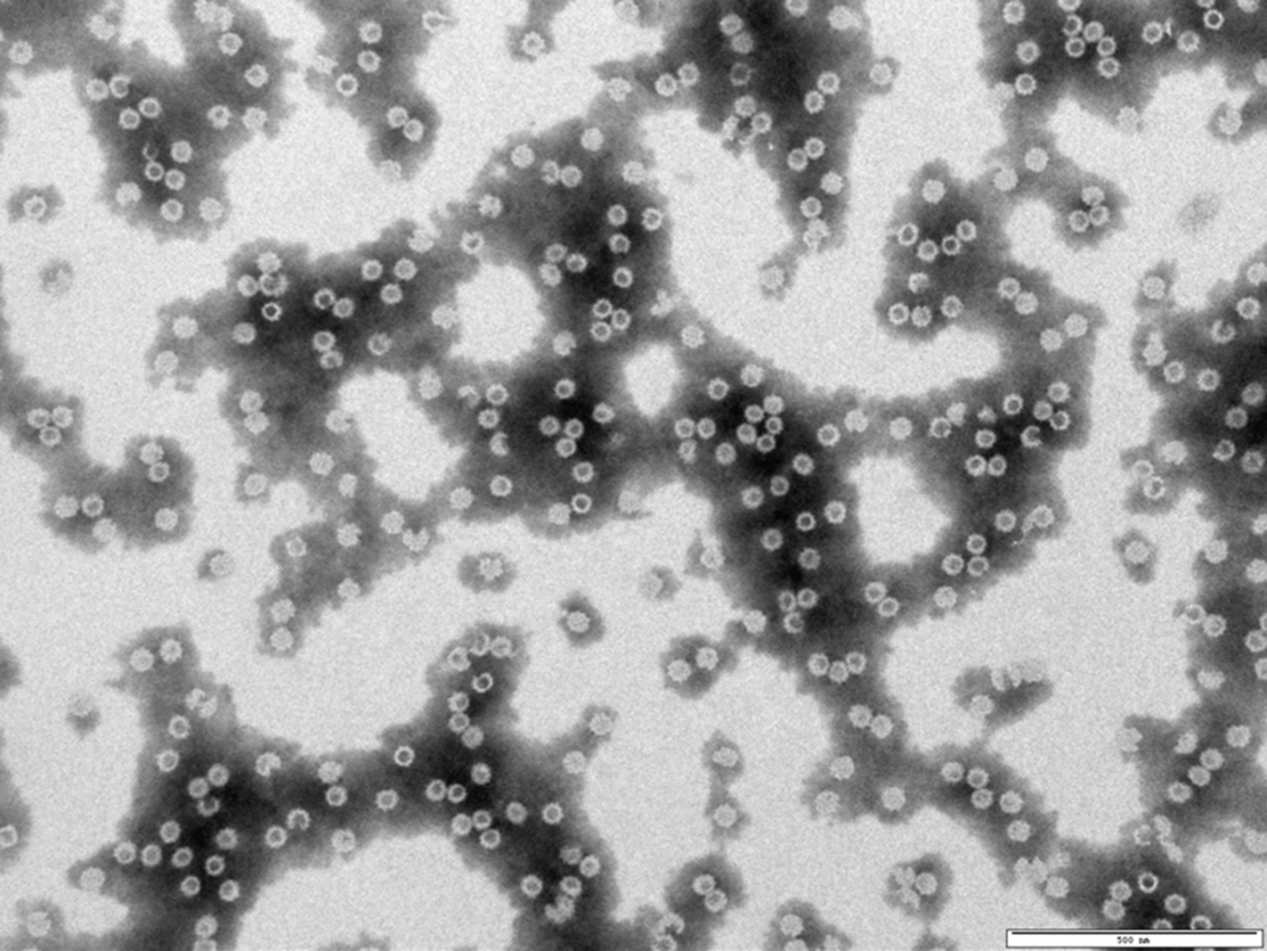
Transmission electron microscopy (TEM) image of virus-like particles (VLPs) assembled from purified IBDV VP2 protein in phosphate-buffered saline (PBS), confirming spherical particle formation. Scale bar = 500 nm.

VP2 is the major outer capsid protein of IBDV, accounting for approximately 51% of the total capsid mass and exhibiting strong immunogenicity (21). Structurally, VP2 comprises three distinct domains the base, shell, and protrusion domains (**Figure 3**) (22) with the protrusion domain containing a hypervariable region that mediates antigenic diversity and elicits virus-neutralizing antibodies. Owing to its high structural stability, antigenic sensitivity, and specificity, VP2 represents a leading target for rational vaccine design (23). Recombinant VP2 has been successfully expressed in multiple heterologous systems, including bacteria (21), yeast (24), baculovirus (25), and plant (26). Notably, recombinant VP2 can self-assemble into virus-like particles (VLPs) that closely mimic the native virion architecture, enhancing its vaccine potential (**Figures 3**, **4**) (22).

A vaccine is a biological immunogen that induces active acquired immunity against a specific disease, typically comprising a weakened or inactivated pathogen, its toxins, or selected surface antigens that mimic the causative microorganism and stimulate protective immune responses.

Vaccination remains the primary strategy for preventing and controlling vvIBDV infections. In China, vvIBDV vaccination is mandatory, particularly in large-scale intensive poultry production systems. Nevertheless, currently available vaccines require substantial improvement. The extensive use of traditional intermediate and hot vaccine strains poses notable biological safety risks (27). Moreover, most vaccines are derived from non-domestic strains, which may exhibit incomplete antigenic matching and consequently confer suboptimal protective efficacy against circulating vvIBDV (28).

The emergence and widespread circulation of highly pathogenic vvIBDV and the novel variant infectious bursal disease virus strain (nVarIBDV) present significant challenges for the effective control of IBD. Notably, nVarIBDV can partially evade immunity induced by existing vvIBDV vaccines, as vvIBDV-derived antisera exhibit limited neutralizing activity against this varIBDV. Consequently, there is an urgent need to develop next-generation vaccines capable of providing broad and robust protection against both emerging variants and highly pathogenic IBDV strains (29, 30).

In addition to viral evolution and antigenic mismatch, several immunological challenges complicate effective control of IBDV. IBDV exhibits a marked tropism for immature IgM^+^ B lymphocytes within the bursa of Fabricius, resulting in lymphoid depletion, follicular atrophy, and long-term immunosuppression that compromises both humoral and cellular immune competence (31). This virus-induced immunosuppression increases susceptibility to secondary infections and reduces responsiveness to other routine vaccinations in commercial poultry flocks. Furthermore, high levels of maternally derived antibodies (MDA) can neutralize live-attenuated vaccine strains, thereby interfering with early vaccination efficacy and creating a narrow immunization window in young chicks (32). The emergence of vvIBDV and nVarIBDV strains with mutations in the VP2 hypervariable region further complicates vaccine-induced cross-protection, as existing vaccines may elicit insufficient neutralizing breadth (33).

Vaccine development against pathogenic microorganisms employs diverse strategies determined by pathogen biology, disease pathogenesis, and practical deployment considerations. Common approaches include live-attenuated, inactivated, and subunit vaccines, each offering distinct advantages and limitations in safety, immunogenicity, and manufacturing (34).

Live vaccines against IBD are derived from attenuated virulent IBDV strains and are categorized as mild, intermediate, or hot based on residual virulence. Mild vaccines offer improved safety but provide limited protection against vvIBDV, whereas intermediate and hot vaccines confer stronger immunity but may cause moderate to severe bursal lesions and immunosuppression (35). Additionally, conventional live vaccines can spread horizontally or vertically and may revert to virulence, compromising biosafety. Consequently, these limitations prevent complete and reliable protection against IBDV in poultry populations (36).

Inactivated, or killed, vaccines consist of non-replicating inactivated whole-virus antigens produced by chemical or physical method, such as formalin or heat treatment (37, 38). Because these vaccines do not replicate in the host, their immunogenicity relies heavily on the use of adjuvants and the administration of multiple booster doses to achieve adequate and sustained immune responses (39). However, the requirement for repeated individual injections substantially increases production and labor costs, limiting their practical application primarily to valuable breeder and parent stock. In poultry production, inactivated vaccines are routinely administered to chickens around 10 days of age as part of structured immunization programs (40).

Inactivated IBD vaccines can elicit inflammatory responses and induce IBDV-specific T-cell immunity in vaccinated birds (35). Although generally safe, their protective efficacy is inferior to that of live-attenuated and VLPs based subunit vaccines, as they typically induce only transient cellular immune responses and confer limited protection against infection and disease (41, 42).

Subunit vaccines consist of defined viral particles capable of eliciting protective immune responses without the use of whole pathogens. This approach is cost-effective and relies on purified fractions or synthetic peptides representing immunogenic proteins of the microorganism (43). Owing to their high purity, structural stability, and favorable safety profile, subunit vaccines minimize biosafety risks while allowing precise antigen design and standardized manufacturing, making them attractive platforms for modern vaccine development (44).

Virus-like particles (VLPs) are non-infectious nanostructures that self-assemble through the expression of viral structural proteins, including capsid, core, or envelope components (45). The successful development of IBDV-targeting VLPs vaccines further supports the translational potential of VLP platforms in poultry viral diseases, demonstrating improved Immunogenicity and structure mimicry compared to conventional systems (46). In IBDV, the VP2 protein contains highly conformational neutralizing epitopes that can self-assemble into VLPs, closely mimicking the native viral capsid both structurally and immunologically (47). In **Figure 4**, the virus-like particles formed by the purified IBDV VP2 protein captured by transmission electron microscopy in our laboratory are presented. VP2-derived capsid-like particles have demonstrated strong immunogenicity and protective efficacy across multiple viral systems (21). Importantly, correctly folded VP2 can form virus-like or subvirus-like particles that elicit robust protective immune responses without viral replication (15, 26, 45). Compared with conventional vaccines, VLPs-based vaccines require lower antigen doses to elicit robust protective immune responses. Moreover, the production of immunogenic capsid-like particles provides a simplified and cost-effective strategy for developing efficacious vaccines against IBDV (48, 49).

Adjuvants are substances, compounds, or complexes that increase the intensity and endurance of a specific immune response to antigens while causing low toxicity or long-term immunological effects (49). Conventional vaccine/adjuvant formulations, including aluminum salts and oil-emulsion adjuvants, primarily stimulate antibody-mediated responses but often fail to induce robust cytotoxic T-cell activity or durable mucosal immunity. Moreover, some oil-based adjuvants may cause local tissue reactions and inconsistent antigen release kinetics (50). These limitations highlight the need for advanced immunomodulatory platforms capable of enhancing antigen presentation, promoting balanced Th1/Th2-type responses, and inducing long-lasting protective immunity against diverse IBDV genotypes.

Nanotechnology-based adjuvants have emerged as promising strategies to address these immunological constraints. Nano-adjuvant systems improve antigen stability, enable controlled release, enhance uptake by dendritic cells and macrophages, and promote lymph node targeting, thereby strengthening both innate and adaptive immune activation (31). In poultry models, nanoparticle-formulated IBDV antigens have demonstrated improved neutralizing antibody titers, enhanced IFN-γ and IL-1β expression, and superior protection against bursal damage following challenge (51). By mimicking pathogen-associated structural features and facilitating efficient antigen delivery, nano-adjuvanted vaccines offer a rational next-generation approach for overcoming immunosuppression, maternal antibody interference, and antigenic variability associated with IBDV.

Nano-adjuvant systems directly address major barriers created by IBDV immunopathogenesis in three ways. First, nanoparticle and nanovesicle delivery improves antigen protection, uptake by antigen-presenting cells, and trafficking to lymphoid sites, promoting stronger germinal center reactions and more consistent antibody quality even when bursal function is compromised early in life (51). Second, nano-adjuvants can be engineered to bias immunity toward balanced humoral and cellular responses by co-delivering PRR agonists (e.g., TLR ligands) with antigen, thereby enhancing innate activation and antigen presentation needed for effective T-cell help and durable memory (51). Third, nano-adjuvants enable dose-sparing and reduce reliance on “hot” live vaccines, which can themselves contribute to bursal lesions and immunosuppression; instead, non-replicating nano-formulated antigens (e.g., VP2 nanoparticle display platforms) can generate protective immunity while minimizing vaccine-associated tissue damage (52).

**Figure 5:**
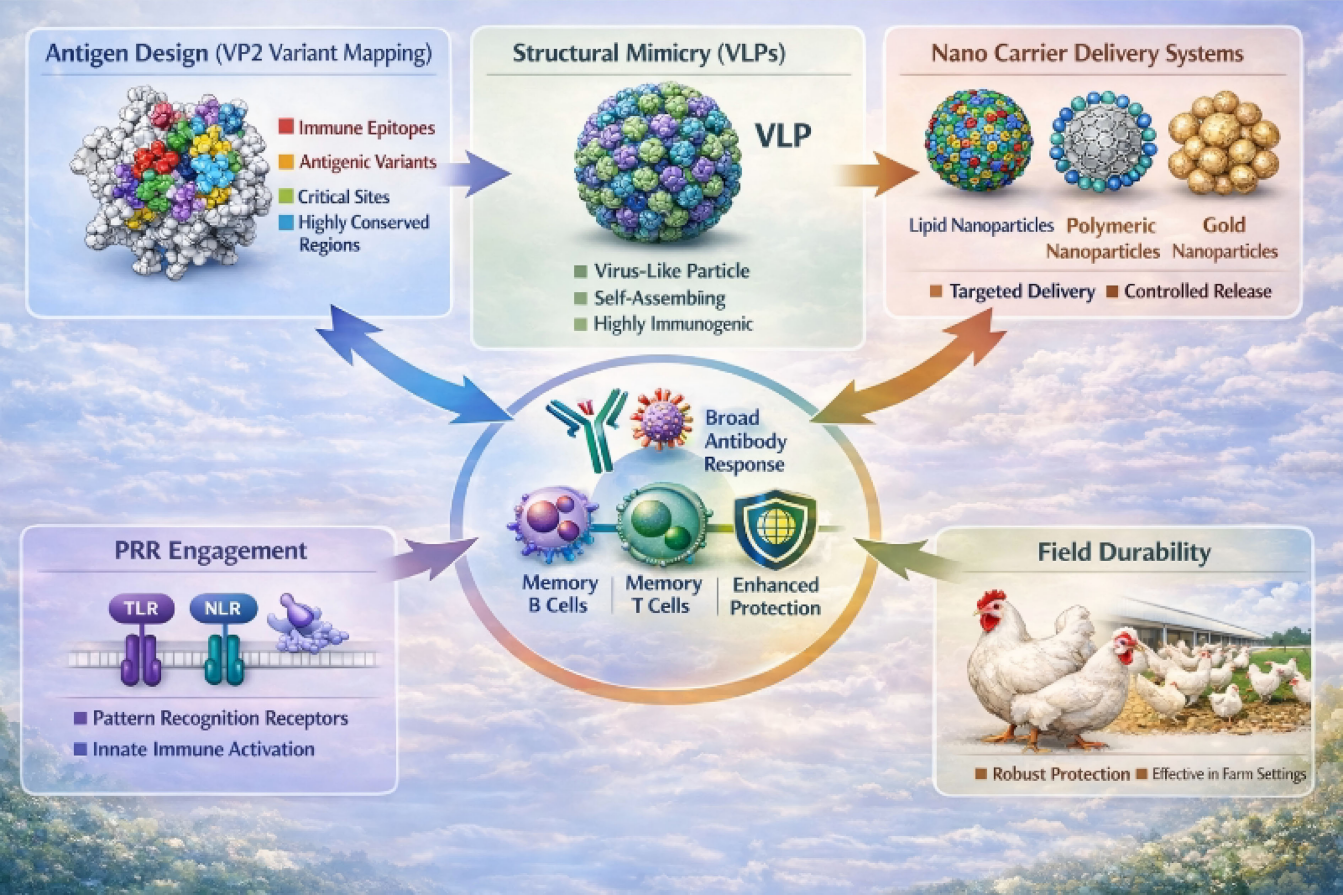
Integrated nano-adjuvant strategy for broad IBDV protection.

Notably, the dose-sparing capacity of nano-adjuvant systems has been quantitatively demonstrated across multiple vaccine platforms. For example, lipid nanoparticle (LNP)-formulated mRNA vaccines have shown that protective immune responses can be achieved with significantly lower antigen doses compared with conventional formulations, with reductions of up to 10-100-fold while maintaining or enhancing neutralizing antibody titers and T-cell responses (53, 54). In veterinary and avian systems, nanoparticle-adjuvanted vaccines, including chitosan and nanoemulsion-based formulations, have also shown significant antigen dose reduction while maintaining protective antibody titers and cellular immune responses (55, 56). These findings highlight that nano-adjuvants not only enhance immunogenicity but also substantially reduce antigen requirements, improving vaccine scalability and cost-effectiveness for large-scale poultry immunization programs. Consistent with this rationale, recent studies show that nanoparticle delivered VP2 DNA and self-assembling VP2 nanoparticle platforms can induce strong immune responses and protection against antigenically divergent nVarIBDV, supporting nano-adjuvants as practical tools to overcome immunosuppression, antigenic mismatch, and early-life vaccination constraints (52) (**Figure 5**).

**Figure 6:**
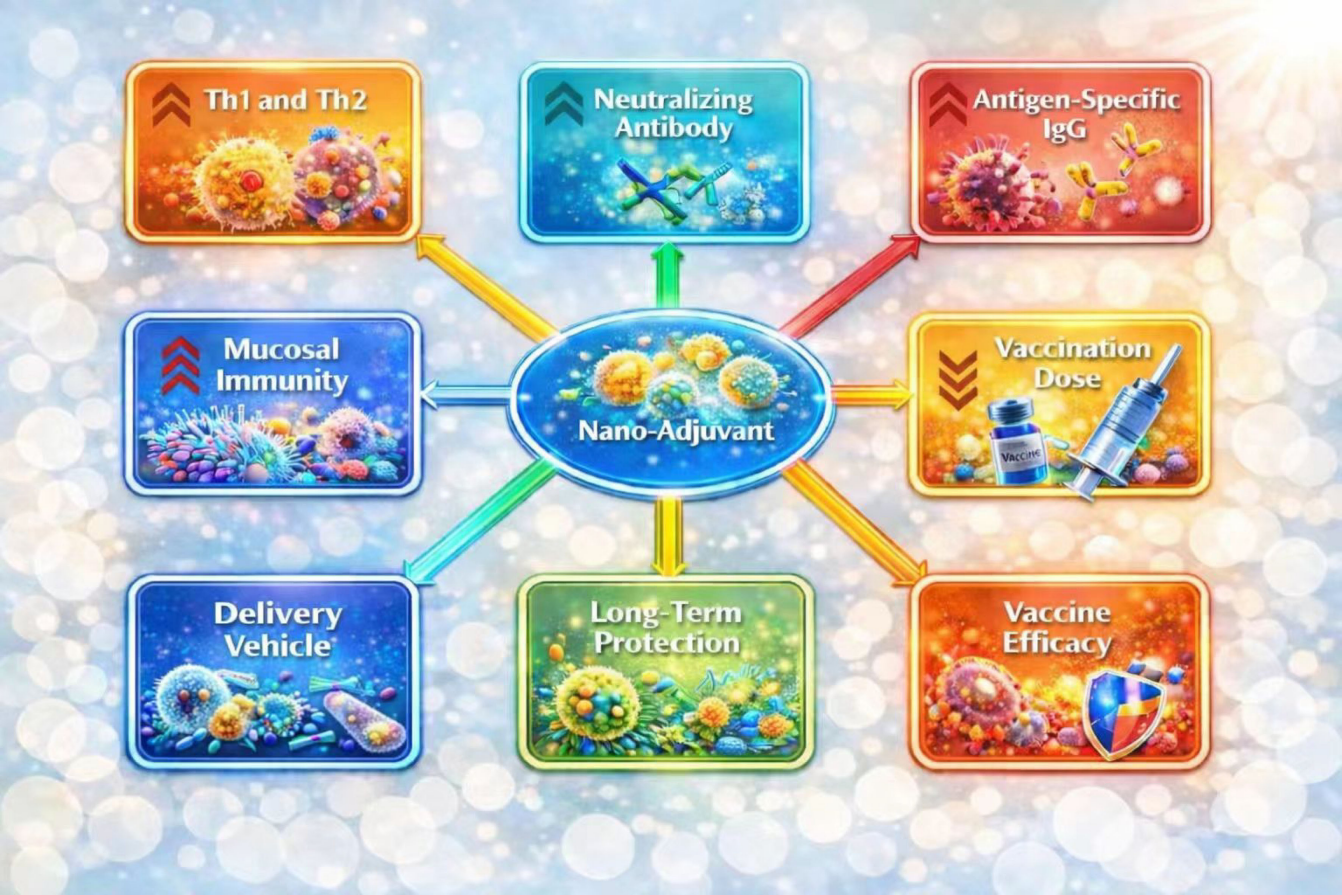
Key advantages of nano-adjuvant systems in vaccine development (57).

**Figure 7:**
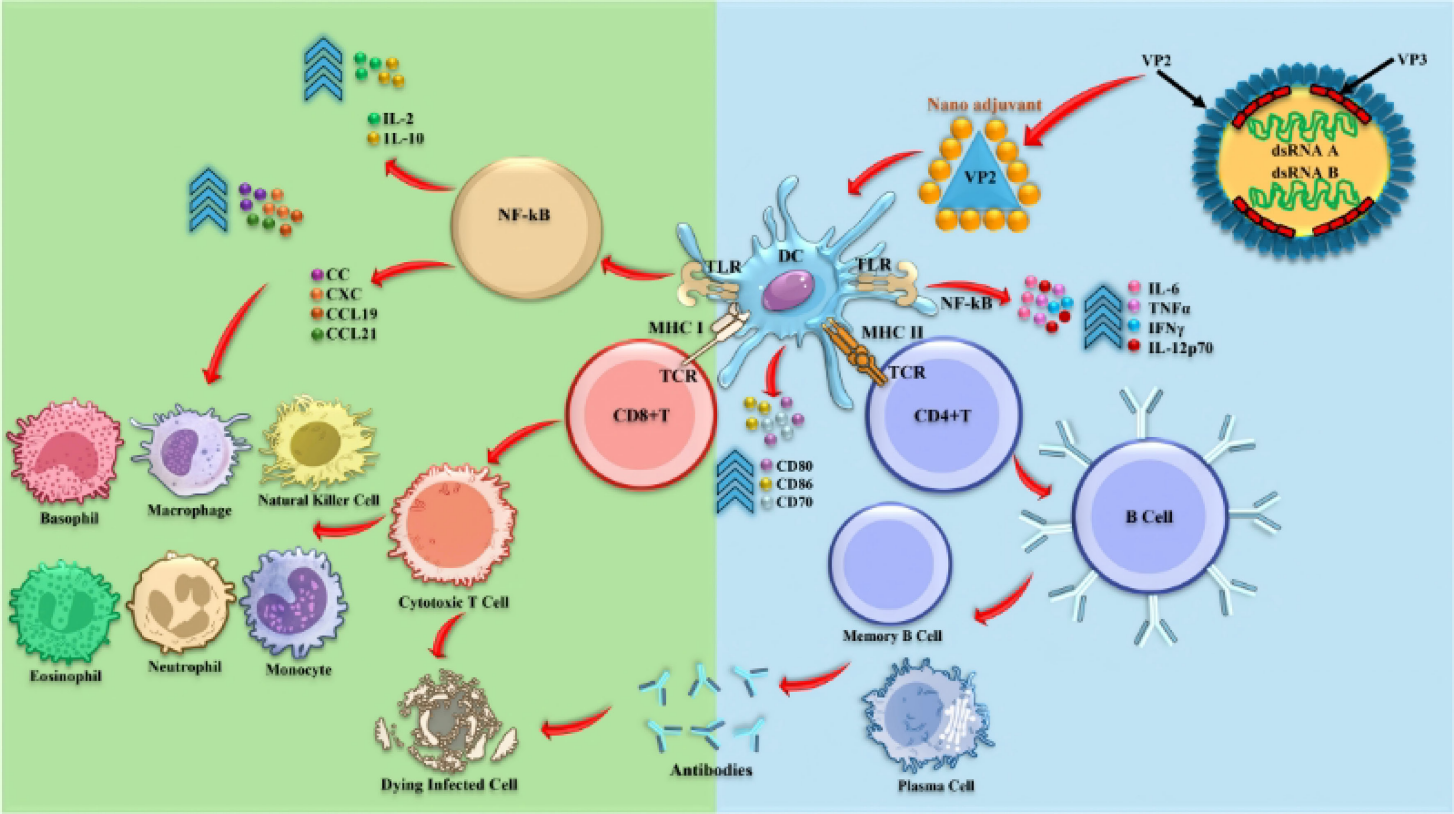
Schematic illustration of the nano-adjuvant–formulated IBDV VP2/VLP complex and its role in enhancing immune responses.

**Figure 8:**
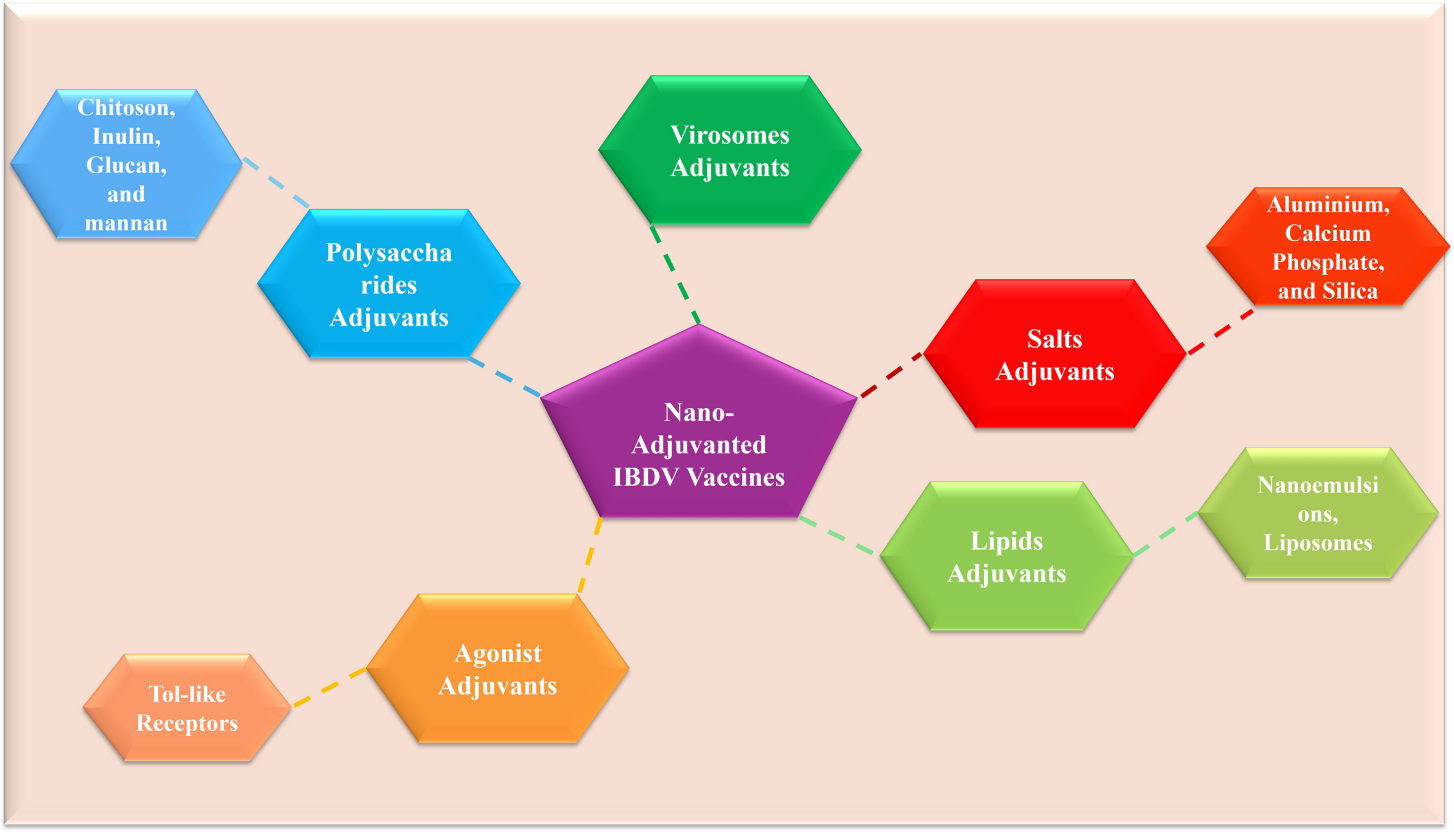
Classification of nano-based adjuvant systems (58).

Current nano-adjuvant systems exert significant immunomodulatory effects in infectious viral diseases (**Figure 6**) (57). Mechanistically, nano-adjuvant-formulated viral antigens, including VP2-based VLPs, enhance immune responses by coordinating innate and adaptive immunity through improved antigen uptake, dendritic cell activation, and cytokine induction (**Figure 7**). Based on their composition and functional properties, nano-adjuvants can be broadly categorized into biomolecule-based systems and inorganic or salt-based platforms (**Figure 8**) (58), each exhibiting distinct immunostimulatory profiles. A comparative role of these nano-based adjuvants in immune modulation is summarized in **Table 1**. Despite their promising immunological advantages, important safety considerations remain, particularly regarding biodegradability, long-term tissue accumulation, and potential residue concerns in food-producing animals (59). Therefore, rigorous optimization of physicochemical characteristics and comprehensive regulatory assessment are critical to ensure the safe and effective integration of these systems into poultry vaccination programs.

**Table 1:** Nano-based adjuvant enhances immune response.

Nano-based adjuvant	Role in immunity	References
Aluminum	Induces IL-1, IL-4, IL-5, IL-10, IL-18, IL-33, IL-1β IgE, IgG1 CD4^+^ T, and Th2	(50, 57–60)
Calcium phosphate	Induces Th1, Th2, and IgG1	(61, 62)
Silica	Provokes IL-4, IFN-γ, IL-6, MHC II, CD86, Th1, and Th2	(63–65)
Liposomes	Activates IFN-γ, TNF-α, IL-2, CD4^+^ T, and IgG	(66)
MF59	Stimulates CCL2, CCL4, CCL5, CXCL8, Th1, and Th2	(64, 65)
AS03	Elevates CCL2, CCL3, IL-6, CXCL1, Th1, and Th2	(65, 66)
Chitosan	Enhances Th1, Th2, IgA, and IgG	(67, 68)
Mannan	Boosts IFN-γ, Th1, and CD169^+^	(69, 70)
Inulin	Triggers CD4^+^ T, CD8^+^ T, and TNF-α	(71)
Glucans	Activates CD4^+^ T, CD8^+^ T, and IL-1	(72–74)
TLRs	Boosts IL-12, IL-6, IL-1β, IL-10, TNF-α, IFN-α, IFN-β, and IFN-γ	(50, 72–75)
VLPs/Virosomes	Stimulates CD4^+^ T, CD8^+^ T, Th1, Th2, CTLs, IgA, and IgG	(76–78)

This review investigates immunological innovations underlying nano-adjuvanted IBDV vaccines. As well as assess how adjuvants enhance potency, protection, and immune responses, while exploring regulatory considerations, emerging delivery approaches, and comparative performance. These advances have led to the development of next-generation IBDV vaccination approaches with enhanced safety, reliability, and impact on the global poultry industry and animal health.

## Salt adjuvant systems

### Aluminum

Aluminum adjuvants, the first adjuvants approved by the U.S. Food and Drug Administration, remain the most widely used adjuvants globally and are central to large-scale vaccination programs. These adjuvants enhance immune responses by facilitating the slow and sustained release of antigens, promoting their uptake, processing, and presentation by antigen-presenting cells to T lymphocytes via major histocompatibility complex molecules. Aluminum salts exhibit distinct immunological properties, including IL-4-dependent induction of IgE responses, while functioning independently of IL-1, IL-18, and the adaptor molecule MyD88 (50, 58). Insoluble aluminum salts preferentially drive CD4^+^ T cells toward Th2-type polarization (62). Conversely, alum displays anti-inflammatory activity by enhancing IL-10 production from macrophages and dendritic cells, thereby suppressing antigen-specific Th1 responses (79). Additionally, aluminum may itself exhibit antigenic properties and enhance antigen presentation by antigen-presenting cells (80).

### Calcium phosphate

Biocompatible calcium phosphate (CaP) nanoparticles have emerged as promising vaccine adjuvants due to their capacity to induce balanced Th1 and Th2 immune responses and reduced antigen requirement to reach protective titers (81). Acting as both carriers and immunostimulatory agents, CaP nanoparticles can activate Th1 responses while facilitating antigen delivery through encapsulation or passive adsorption at various stages of particle formation (82, 83). When combined with Toll-like receptor (TLR) ligands and viral antigens, CaP nanoparticles enhance the maturation of human antigen-presenting cells, as indicated by increased expression of co-stimulatory molecules and secretion of proinflammatory cytokines, which collectively promote the expansion of virus-specific T cells (84). Supporting evidence from murine models demonstrates that intramuscular administration of CaP nanoparticles enhances hepatitis B surface antigen expression, antigen-specific T cell proliferation, and IgG1 antibody responses (85). Additionally, CaP offers physicochemical advantages as a vaccine carrier; its pH-dependent solubility enables controlled release of encapsulated agents, including plasmid DNA, thereby optimizing antigen presentation and immunogenicity (63). These properties position CaP nanoparticles as versatile and effective platforms for next-generation vaccine development.

### Silica

Vaccines against viral pathogens often consist of large bioactive molecules, such as proteins or DNA, which require specialized delivery systems to achieve effective immunization. Mesoporous silica nanoparticles (MSNs) possess ideal characteristics for this purpose, including high colloidal stability, large specific surface area, extensive surface functionalization capacity, and substantial pore volume, while their amorphous silica composition allows gradual biodegradation *in vivo*, ensuring biocompatibility. MSNs can modulate immune responses by delivering cytokines; for example, large-pore MSNs (30 nm) efficiently loaded and delivered IL-4, promoting M2 macrophage polarization *in vivo*, and exhibited superior protein-loading capacity compared with conventional small-pore MSNs (3.2 nm) (64). Furthermore, mesoporous silica rods (MSRs) can self-assemble into three-dimensional microporous scaffolds that support host immune cell infiltration and activation. Administration of MSR-based vaccines enhanced systemic Th1 and Th2 antibody responses and cytotoxic T-cell activity, while dendritic cells were recruited into scaffold pores through the release of inflammatory signals and adjuvants such as single-stranded DNA and CpG-oligodeoxynucleotides, highlighting the potential of MSNs and MSRs as versatile platforms for next-generation vaccine delivery (65).

Mesoporous silica can enhance adjuvant immunological function when combined with other inorganic vaccine carriers. For example, SiO_2_ nanoparticles with layered double hydroxide core-shell structures efficiently delivered DNA vaccines and activated macrophages, significantly increasing IFN-γ, IL-6, MHC II, and CD86 expression. This led to enhanced immune responses in mice, including T-cell proliferation and a shift toward Th1 polarization, highlighting their potential as multifunctional vaccine adjuvants (86).

The physicochemical properties of mesoporous silica rods (MSRs) can be tailored by conjugating diverse surface ligands. A pH-sensitive co-carrier system was developed using mesoporous silica as the core, coated with a metal organic framework and adsorbed CpG nucleic acids (87). This design addresses challenges including poor antigen endocytosis, limited immunostimulatory activity, and antigen degradation, while its pH-responsive behavior stabilizes the antigen and enhances controlled cellular release, improving overall vaccine efficacy.

## Lipid-based adjuvant system

### Nano emulsions

Nanoscale emulsions, or nanoemulsions, are heterogeneous systems composed of two immiscible liquids, in which one phase is finely dispersed within the other, typically forming droplets 20–200 nm in diameter. These dispersions can exist as water-in-oil (W/O) or oil-in-water (O/W) systems, with their physical stability determined by the careful selection of components and preparation methods (88). Nanoemulsions possess multiple physicochemical properties favorable for biomedical applications, including high surface area, enhanced solubilization of hydrophobic compounds, and versatility across administration routes such as parenteral, dermal, oral, ocular, and nasal delivery (89–92). In vaccine development, these lipid-based colloidal systems have been employed as adjuvants in both human and veterinary vaccines, facilitating antigen presentation, prolonging antigen release, and protecting antigens from rapid degradation or clearance, thereby enhancing immunogenicity and overall vaccine efficacy (86, 87). Their tunable composition and nanoscale size make nanoemulsions highly adaptable platforms for next-generation immunization strategies.

The PF3 nano-adjuvant, prepared via microfluidization by combining saponin (Ginsenoside Rg1) with an oil-in-water nanoemulsion, enhances both neutralizing antibody titers and antigen-specific CD4^+^/CD8^+^ T-cell responses, unlike conventional aluminum adjuvants, which primarily elicit humoral immunity. PF3 demonstrates superior immunostimulatory potency, improved safety, and high physicochemical stability, making it a promising adjuvant for recombinant protein vaccines (66).

### Liposomes

Liposomes are spherical vesicles composed of one or more lipid bilayers that serve as versatile carriers for therapeutic agents, including antigens and immunomodulators, highlighting their potential as vaccine adjuvant systems. Vaccines formulated with lyophilized protein antigens and cationic liposomes elicited markedly elevated IFN-γ, TNF-α, and IL-2 production in antigen-specific CD4^+^ T cells of immunized mice, demonstrating enhanced cellular immune responses (93).

Covalent conjugation of antigens to liposomes enhances the immunogenicity of lipid nanoparticle-based vaccines by strengthening humoral immune responses. Immunization with covalently linked MD39 trimers, a stabilized trimer variant, significantly increased germinal center formation, antigen-specific T follicular helper cell levels, and serum MD39-specific IgG compared with soluble MD39 trimers (94). Covalent attachment preserves the structural integrity of the trimer, promoting the generation of targeted antibodies. Moreover, high-density, well-organized presentation of trimers on liposomes improves antigen orientation and eliminates responses against extraneous regions, such as the C-terminal His tag, enhancing the specificity and quality of the humoral response relative to soluble trimers (95).

In chickens, immunization with Monophosphoryl lipid A (MPLA)-adjuvanted virosomes or recombinant vaccine platforms expressing IBDV structural proteins (VP2 or VP3) elicits robust antigen-specific cytotoxic T lymphocyte responses, strong neutralizing antibody titers, and confers protection against bursal atrophy and immunosuppression following virulent IBDV challenge (36, 91). MPLA enhances dendritic cell activation through Toll-like receptor 4 (TLR 4) signaling, promoting Th1-biased immune responses that are critical for viral clearance (96). Additionally, MPLA in combination with aluminum hydroxide enhances recruitment of monocytes and heterophils, facilitates IgY isotype switching, and increases antibody-secreting cell responses in chickens, contributing to improved protection against vvIBDV strains. These findings highlight the potent immunostimulatory and protective capabilities of MPLA-based adjuvant systems in poultry vaccines (33, 36, 91).

AS03 is an oil-in-water emulsion adjuvant composed of squalene and α-tocopherol that enhances immune responses through activation of innate immune pathways, including recruitment of antigen-presenting cells (APCs), induction of cytokine production, and promotion of antigen uptake and lymph node trafficking (92, 93). Similar to MF59, AS03 stimulates local innate immune activation, thereby enhancing downstream antigen-specific humoral and cellular responses (97).

In avian vaccine research, oil-in-water emulsions comparable to AS03 have demonstrated significant enhancement of IBDV-specific antibody titers, virus-neutralizing activity, and cell-mediated immune responses when formulated with recombinant VP2 or inactivated IBDV antigens (33, 73, 98, 99). These formulations improve protection against very virulent and variant IBDV strains, reducing bursal lesion severity and viral replication. Oil-in-water emulsion-based veterinary vaccines have shown favorable safety and efficacy profiles in poultry, although optimization of emulsion composition and dose is necessary to minimize injection-site reactions and ensure consistent immune potentiation (100–102).

Modern emulsion manufacturing approaches, including systems analogous to AS03, frequently employ phase inversion temperature (PIT) technology, a low energy nanoemulsification method that utilizes intrinsic physicochemical properties of surfactants and oil phases to generate stable emulsions. PIT-based methods enhance scalability, batch consistency, and cost-effectiveness in vaccine production (103–106).

Moreover, montanide adjuvants, including ISA 71, ISA 206, ISA 70, ISA 51, and ISA 720, represent water-in-oil or water-in-oil-in-water nanoemulsion formulations widely employed in veterinary vaccines, including those targeting IBDV. These adjuvant systems enhance antigen persistence at the injection site, stimulate strong and long-lasting antibody responses, and improve protection against both classical and vvIBDV strains. Several recent studies have demonstrated that Montanide-based IBDV vaccines significantly reduce mortality, viral shedding, and bursal damage compared with non-adjuvanted formulations (105, 106).

## Agonist adjuvant systems

Agonist adjuvants, also termed immunostimulants, constitute a critical class of danger signal molecules that enhance vaccine efficacy by promoting the maturation and activation of antigen-presenting cells through specific receptor engagement. These agents function by activating pattern recognition receptors (PRRs), thereby inducing distinct cytokine profiles that shape the magnitude, quality, and polarization of adaptive immune responses. Based on ligand specificity and structural features, PRRs are broadly categorized into Toll-like receptors (TLRs), C-type lectin receptors, nucleotide-binding oligomerization domain-like receptors (NLRs), retinoic acid-inducible gene I-like receptors (RLRs), and absent in melanoma 2-like receptors (ALRs) (107, 108). Among these families, TLRs are the most extensively characterized and exhibit the widest ligand recognition spectrum. TLR1, TLR2, TLR5, and TLR6 are localized on the cell surface and primarily detect extracellular pathogens, whereas TLR3, TLR7, TLR8, and TLR9 reside within endosomal compartments and recognize viral nucleic acids and endogenous danger signals. Notably, TLR4 uniquely signals from both the plasma membrane and endosomes, enabling context-dependent immune activation (50, 109).

Encapsulation of Nexavant (NVT), a double-stranded RNA-based TLR3 agonist, within lipid nanoparticles (LNPs) markedly enhanced cellular immune responses by improving intracellular delivery to target cells. Importantly, the magnitude of the *in vivo* immune response depended on the composition of the ionizable lipids, underscoring the critical role of lipid selection in formulation design. When combined with optimized ionizable lipids and used as an adjuvant for peptide-based vaccines, NVT/LNP outperformed conventional adjuvants and conferred robust therapeutic efficacy in viral infection models. Collectively, these findings highlight NVT/LNP as a potent and versatile adjuvant platform for vaccines aimed at inducing strong cellular immunity (110).

Recent studies indicate that nanoparticle-based adjuvant systems, particularly those incorporating TLR agonists, significantly enhance dendritic cell maturation, and antigen uptake, and subsequent cell-mediated immunity (111). Emerging formulations, including CpG-loaded nanoparticles and emulsions, represent a paradigm shift from traditional depot-based adjuvants toward targeted engagement of pattern recognition receptors on antigen-presenting cells, thereby promoting Th1-biased and durable memory immune responses (83).

Current experimental evidence demonstrates the efficacy of hybrid and polymeric nanoparticle adjuvant systems incorporating TLR agonists. A hybrid aluminum-emulsion nanoparticle co-delivered with CpG, a TLR9 agonist, markedly enhanced dendritic cell uptake, upregulated co-stimulatory molecules and MHC II expression, promoted lysosomal antigen escape, and induced robust antigen-specific CD4^+^ and CD8^+^ T-cell responses that significantly exceeded those elicited by either component alone (112). Similarly, polymeric nanoparticles encapsulating the TLR7 agonist gardiquimod sustained dendritic cell and B-cell activation within draining lymph nodes, promoted strong germinal center formation with increased B-cell receptor diversity, and generated potent antigen-specific CD8^+^ cytotoxic T lymphocytes, including lung-resident memory T cells, together with cross-reactive broadly neutralizing antibody responses (113).

**Figure 9:**
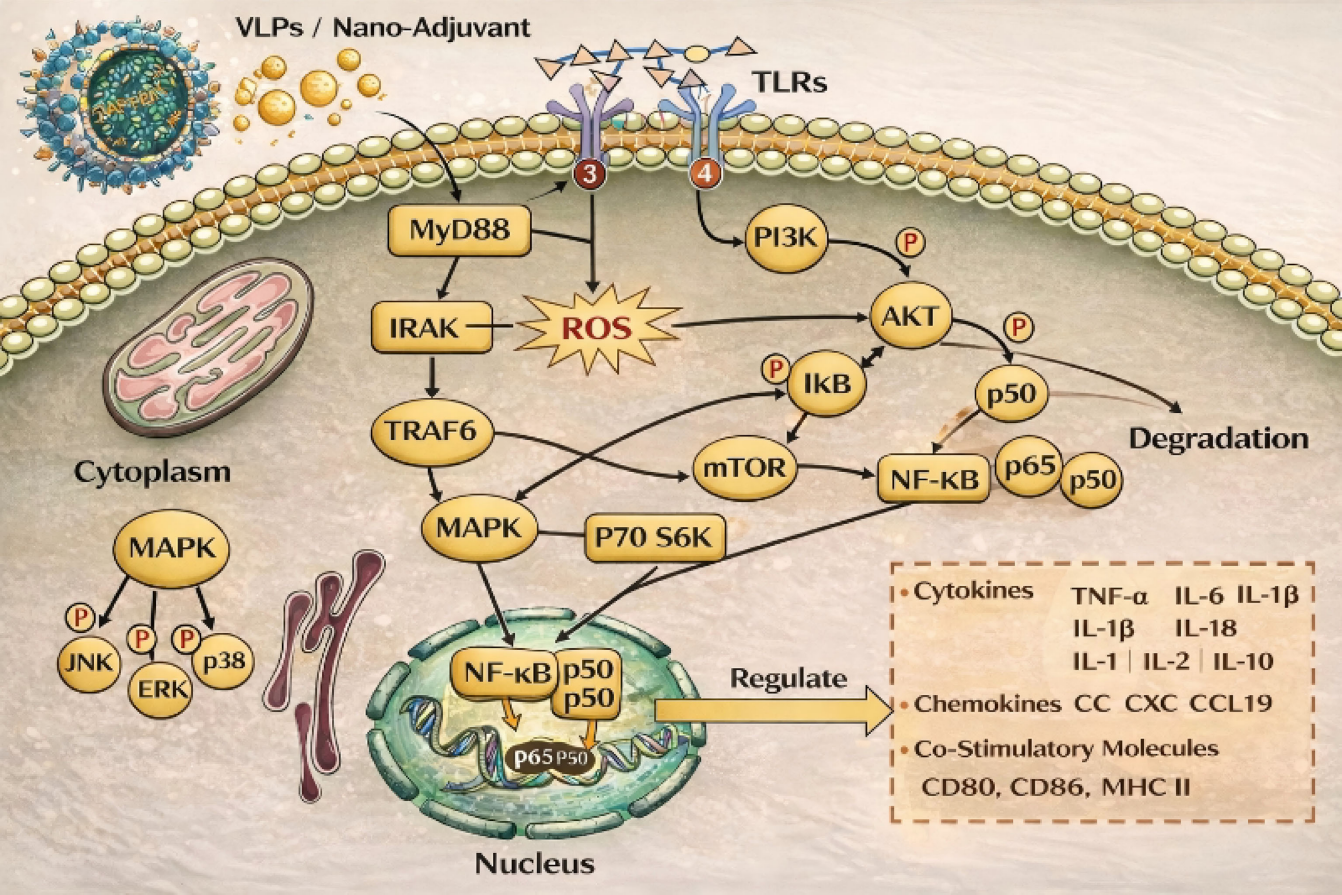
Signaling pathways underlying macrophage activation induced by VLPs and nano-based adjuvants (70).

Specifically, nano-based adjuvant activates macrophages or dendritic cells through the interaction of TLR 4, induce activation of the MAPK and NF-κB pathways, and eventually produce cytokines, chemokines, and co-stimulatory molecules, and stimulate degradation of the IκBα (NF-κB inhibitor) IκBα (75). Similar Immunomodulatory effects through NF-kB, STAT3, and apoptosis-related pathways have also been reported for natural bioactive compounds, such as C-phycocyanin, which enhances immune cell proliferation without cytotoxicity (114). **Figure 9** shows the relevant pathway underlying the immunomodulatory effect of nano-adjuvants on macrophage activation (70).

## Polysaccharide nano-adjuvant

### Chitosan

Chitosan is a naturally derived polysaccharide with excellent biocompatibility and immunoadjuvant potential. Compared with alum, chitosan has been reported to function as a superior adjuvant for hepatitis A vaccination while maintaining comparable formulation characteristics (115). Notably, chitosan can promote a balanced Th1/Th2 immune response. Alginate-coated chitosan nanoparticles further enhance vaccine immunogenicity, achieving complete seroconversion, elevated pathogen-specific antibody titers, and increased splenocyte proliferation in immunized animals following experimental vaccination protocols studies (115).

Chitosan’s strong cationic nature confers high affinity for nucleic acids, enabling its effective application as a carrier for nucleic acid and peptide antigens. Biotinylated chitosan nanoparticles encapsulating plasmid DNA selectively target dendritic cells and enhance mucosal IgA and systemic IgG responses, supporting their potential utility in low-dose vaccine development (116).

Trimethyl chitosan conjugated with peptide antigens enhances serum antibody responses, representing a promising strategy for developing effective vaccine candidates (117). Precise control of nanoparticle size is essential to ensure robust and stable immune activation in such vaccine delivery systems (118).

### Mannan

Mannan, a polysaccharide composed of β (1→4)-linked D-mannose units, functions as a potent immunomodulatory adjuvant by enhancing antigen presentation, promoting dendritic cell maturation, and activating complement pathways through interactions with mannan-binding lectin and C-type lectin receptors (119). Fungal-derived mannan induces robust innate immune responses within lymph nodes that depend on dectin-2 expressing CD169^+^ sinus macrophages, noncanonical NF-κB signaling, and interferon pathways. When combined with alum, mannan markedly augments neutralizing antibody production and confers protection against viral infection (120). Similarly, D-galacto-D-mannan functions as an effective vaccine adjuvant by engaging C-type lectin receptors such as Dectin-2, thereby promoting coordinated cellular and humoral immune responses in poultry. Activation of Dectin-2-associated signaling pathways enhances antigen presentation, cytokine production (including IL-1β, IL-6, and IFN-γ), and B-cell activation, contributing to improved protective immunity against viral infectious diseases following vaccination (69, 118, 120). Moreover, MGCP polysaccharides containing mannan and β-glucan differentially regulate immune outcomes, with mannan-enriched formulations promoting regulatory T cell induction and anti-inflammatory effects via dectin-1-COX-2 signaling while limiting IFN-γ driven Th1 differentiation (121), emphasizing the context-dependent immunological versatility of mannan-based adjuvants across diverse vaccine platforms and infectious disease settings.

### Glucan

Glucans are anionic polysaccharides comprising complex, branched polymers of glucose units and represent a versatile class of biocompatible immunomodulators. Similar to chitosan, both α-glucans, such as dextran, and β-glucans exhibit potent immunoenhancing properties with minimal toxicity. Early studies identified dextran sulfate as a promising adjuvant capable of modulating both humoral and cell-mediated immunity, with adjuvant efficacy strongly influenced by molecular weight; high molecular weight dextran sulfate (900 kDa), but not low molecular weight forms (20 kDa), markedly enhanced antibody responses (72). Chemical conjugation of dextran with CpG DNA further expands its utility, promoting robust Th1-polarized CD4^+^ T cell and cytotoxic T lymphocyte responses, reducing immunosuppressive CD11b^+^Gr1^low myeloid-derived suppressor cells, and eliciting pronounced antitumor activity (73). Acetalated dextran, a tunable acid-sensitive derivative, has additionally demonstrated capacity to enhance antigen presentation, CD8^+^ T cell activation, and humoral immunity (74), underscoring its potential as a vaccine adjuvant. In parallel, growing evidence supports the immunostimulatory activity of β-glucans, which can induce trained innate immunity in poultry and enhance resistance to poultry viral diseases through IL-1β dependent and other pro-inflammatory signaling pathways (122–124). In chickens, β-glucan supplementation has been shown to enhance macrophage activation, increase expression of IL-1β, IL-6, and IFN-γ, and improve both humoral and cellular immune responses following IBDV vaccination or challenge, thereby contributing to reduced viral replication and improved bursal protection (125, 126). β-Glucans derived from Grifola frondosa enhance antigen-presenting cell maturation in a dose-dependent manner and significantly augment antigen-specific antibody responses (126).

Moreover, β-glucan–containing polysaccharides, including α (1→3)-, α (1→4)-, and β (1→6)-linked glucans, have been shown to potentiate the various kind of vaccines by enhancing virus-specific antibody production, increasing neutralizing titers, and stimulating cytokine secretion such as IL-1β, IL-6, IFN-γ, and TNF-α in poultry (127). These immunomodulatory effects contribute to improved bursal protection and reduced viral replication following virulent IBDV challenge, highlighting the potential of β-glucans as next-generation adjuvants in vaccine development (128).

## Virus based nano-adjuvant

Virosomes are a virus-mimicking vaccine platform composed of envelope proteins derived from recombinant viruses that self-assemble into virus-like particles (VLPs) (129). These spherical vesicles, ranging from 60 to 200 nm and composed of lipid nanomaterials, are FDA-approved nanocarriers that lack nucleocapsids (130). Functioning as highly effective antigen delivery systems, virosomes elicit robust both antibody-mediated and T cell-mediated immunity comparable to natural infection or potent adjuvants (131). They efficiently deliver antigens into the cytosol of antigen-presenting cells, facilitating processing and presentation that induce cytotoxic T lymphocyte responses (78). A key advantage of virosomes lies in their ability to adsorb antigens onto both surface and internal compartments via hydrophobic interactions, enhancing antigen stability and improving immune activation (83). Furthermore, studies have demonstrated that IBDV VP2 presented as VLPs serves as a potent vaccine candidate, conferring broad protection against both vvIBDV and nVarIBDV strains (30, 132), highlighting the potential of virosome-based platforms in next-generation vaccine design. **Table 2** summarize the comparative assessment of nano-adjuvant systems for IBDV vaccines in poultry.

**Table 2:** Comparative evaluation of nano-adjuvant platforms for IBDV vaccines in poultry.

Platform	Evidence in chicken models	Immunogenicity profile (Th1/Th2)	Manufacturing scalability	Field applicability (poultry)	Cost feasibility	Regulatory readiness	Key advantages	Key limitations	References
IBDV VP2-ferritin protein nanoparticles	Demonstrated protective immune responses against variant IBDV in chickens	Strong humoral response; balanced Th1/Th2 potential	Recombinant protein production; scalable with established expression systems	Primarily injectable	Moderate	Comparable to subunit vaccines	High antigen display density; structural stability	Requires injection; purification cost	(52)
IBDV VP2-based virus-like particles (VLPs)	Proven immunogenic and protective in chickens	Robust antibody responses; supports cellular immunity	Baculovirus, *E. coli*, yeast systems; moderate complexity	Injectable; limited mass spray data	Moderate-High	Veterinary precedents exist	Excellent structural mimicry; strong B-cell activation	Production yield variability; handling logistics	(42)
Lipid nanoparticles (LNPs) delivering IBDV VP2 DNA/mRNA	Recent chicken immunogenicity data	Promotes Th1-biased cellular responses	Advanced formulation; higher CMC complexity	Injectable; cold-chain sensitivity possible	Higher	Emerging in veterinary field	Rapid antigen redesign; strong cellular immunity	Cost-of-goods; regulatory scrutiny	(51)
Polymeric/chitosan nanoparticles	Limited direct poultry viruses challenge data; strong theoretical and mucosal potential	Enhances Th1/Th2 balance; mucosal IgA	Relatively simple fabrication	Compatible with mucosal delivery (spray/oral potential)	Low-Moderate	Favorable if biodegradable	Suitable for mass vaccination; mucoadhesive	Limited poultry virus-specific validation	(133, 134)
Inorganic nanoparticles (e.g., silica, CaP)	No extensive IBDV-specific field validation	Strong APC activation; Th1-skewing possible	Scalable synthesis	Primarily injectable	Moderate	Residue concerns in food animals	Potent immune activation	Biodegradability and tissue accumulation concerns	(133, 135–137)

## Translational considerations for poultry and IBDV vaccines

IBDV specifically targets the bursa of Fabricius, leading to destruction of immature B lymphocytes and long-term impairment of humoral immunity (35). Accordingly, vaccine platforms must restore effective bursa-dependent immune responses while minimizing immunopathology.

The avian innate immune system differs from mammals in pattern recognition receptor (PRR) composition and signaling. Chickens possess unique receptors such as TLR21, which functionally resembles mammalian TLR9 in CpG DNA sensing, and show distinct cytokine response profiles (138). Nano-adjuvant systems designed for poultry should therefore prioritize ligands and delivery strategies compatible with avian-specific PRR engagement.

Maternal-derived antibodies (MDA) remain a major constraint in IBDV vaccination programs. High MDA titers in young chicks can neutralize vaccine antigens and reduce vaccine take, particularly with live or subunit vaccines (138). Optimized nano-formulations may enhance antigen uptake and presentation; however, vaccination schedules must still account for MDA decay kinetics in commercial flocks.

Lipid nanoparticle (LNP)-encapsulated mRNA vaccines protect antigenic payloads within an ionizable lipid bilayer, preventing extracellular antibody binding and enabling intracellular antigen expression following endocytosis, thereby bypassing antibody-mediated neutralization. Once internalized by dendritic cells, antigen is synthesized endogenously and presented via MHC-I and MHC-II pathways, promoting robust cellular and humoral responses independent of extracellular antigen exposure (53). Chitosan-based nanoparticles exhibit strong mucoadhesive and cationic properties, enabling paracellular transport across epithelial barriers and direct uptake by M cells and dendritic cells in mucosal-associated lymphoid tissues (MALT). This allows antigen delivery to immune inductive sites with reduced exposure to circulating antibodies. In addition, chitosan can facilitate endosomal escape via the “proton sponge effect,” enhancing cytosolic antigen delivery and cross-presentation, thereby strengthening T-cell responses even in the presence of high MDA titers (134).

From a deployment perspective, poultry vaccination relies heavily on mass administration methods including spray, drinking water, and in ovo delivery at hatcheries. Therefore, nano-adjuvant platforms must demonstrate compatibility with collective delivery systems rather than solely injectable formats (139).

In commercial poultry production, mass vaccination is predominantly performed via spray, drinking water, or aerosolized delivery systems rather than individual injections. Therefore, the applicability of nano-adjuvant platforms must also be evaluated in the context of mucosal immunization. Recent studies indicate that nano-adjuvants, particularly polymeric and lipid-based nanoparticles, can significantly improve antigen stability and uptake across mucosal surfaces. For example, chitosan-based nanoparticles exhibit strong mucoadhesive properties, enabling prolonged antigen retention on mucosal surfaces and enhanced uptake by microfold (M) cells and antigen-presenting cells in the respiratory and gastrointestinal tracts. This results in improved induction of both systemic IgG and mucosal IgA responses (140).

Similarly, nanoemulsions and LNPs have demonstrated the ability to protect antigens from enzymatic degradation in drinking water and gastrointestinal environments, while facilitating trans-epithelial transport and efficient immune activation (133, 134). Spray-delivered nanoparticle vaccines have also been shown to enhance respiratory mucosal immunity by promoting antigen uptake in the nasal-associated lymphoid tissue (NALT), leading to improved local and systemic immune responses in poultry (141).

Importantly, physicochemical characteristics such as particle size, surface charge, and hydrophobicity critically influence mucosal absorption efficiency. Nanoparticles in the range of 100–300 nm with positive surface charge exhibit enhanced interaction with negatively charged mucosal membranes, improving uptake and immune activation (142). Collectively, these findings highlight that nano-adjuvants are not only compatible with mass vaccination strategies but may also overcome key limitations of conventional mucosal vaccines, including poor antigen stability and low immunogenicity.

Economic feasibility is a critical determinant of translational success. Poultry vaccination operates under strict cost-per-dose limitations, requiring scalable manufacturing processes, minimal cold-chain dependence, and formulation stability under field conditions (139).

Finally, regulatory approval for food-producing animals requires comprehensive evaluation of nanoparticle biodistribution, biodegradability, tissue accumulation, and potential residue concerns. Recent veterinary regulatory discussions emphasize nanoparticle-specific safety assessment prior to field implementation (136).

## Conclusion and future perspectives

IBDV continues to pose a major threat to global poultry production due to its high virulence, rapid transmission, and the emergence of variant strains that partially evade immunity induced by conventional vaccines. Traditional live-attenuated, inactivated, and subunit vaccines, although widely used, exhibit limitations including incomplete protection, safety concerns, antigenic mismatch, and the need for repeated dosing. Advances in nano-adjuvant technology offer a transformative approach to overcome these challenges by enhancing vaccine immunogenicity, stability, and targeted delivery. Nanoformulations, including lipid nanoparticles, polymeric systems, virosomes, mesoporous silica, polysaccharides-based platforms, and emulsions have demonstrated the capacity to elicit robust humoral and cellular immune responses, promote Th1/Th2 balance, facilitate antigen-specific T-cell and B-cell activation, and enable dose-sparing strategies. Agonist-based nano-adjuvants, particularly those incorporating TLR ligands, further augment dendritic cell maturation, antigen presentation, and memory immune responses, highlighting their potential in next-generation vaccine platforms.

Looking forward, the rational design of IBDV vaccines should prioritize precise antigen selection, structural mimicry through VLPs or virosomes, and multifunctional nano-adjuvant systems to achieve broad and durable protection against emerging variants such as vvIBDV and nVarIBDV. Integration of advanced formulation strategies including pH-sensitive carriers, covalent antigen conjugation, and combinatorial adjuvant systems can enhance immunogenicity while maintaining safety and scalability. Future research should focus on optimizing nanoparticle physicochemical properties, elucidating mechanisms of immune modulation, and validating efficacy in diverse poultry populations under field conditions. Collectively, these innovations have the potential to revolutionize IBDV vaccination, providing safer, more effective, and globally deployable solutions that safeguard poultry health and production efficiency.
